# Supplementary material for: Quantum Search on Encrypted Data Based on Quantum Homomorphic Encryption
Source: Sci Rep. 2020 Mar 20;10:5135. doi: 10.1038/s41598-020-61791-9 (PMC7083879; doi:10.1038/s41598-020-61791-9)
Supplement: Supplementary file 1 — Supplementary figure. [file 41598_2020_61791_MOESM1_ESM.pdf]

Quantum Search on Encrypted Data Based on Quantum Homomorphic Encryption

Qing Zhou[1], **Songfeng Lu**[1,2,*], **Yongquan Cui**[1,*], **Li Li**[3], and **Jie Sun**[1]

[1]School of Computer Science and Technology, Huazhong University of Science and Technology, Wuhan 430074, China
[2]Shenzhen Huazhong University of Science and Technology Research Institute, Shenzhen 518063, China
[3]College of Mathematics and Statistics, Shenzhen University, Shenzhen 518060, China
[*]corresponding author: Songfeng Lu (lusongfeng@hotmail.com) or Yongquan Cui (yqcui1977@hust.edu.cn)

January 28, 2020

Supplement: The quantum homomorphic search circuit for two-qubit encrypted states

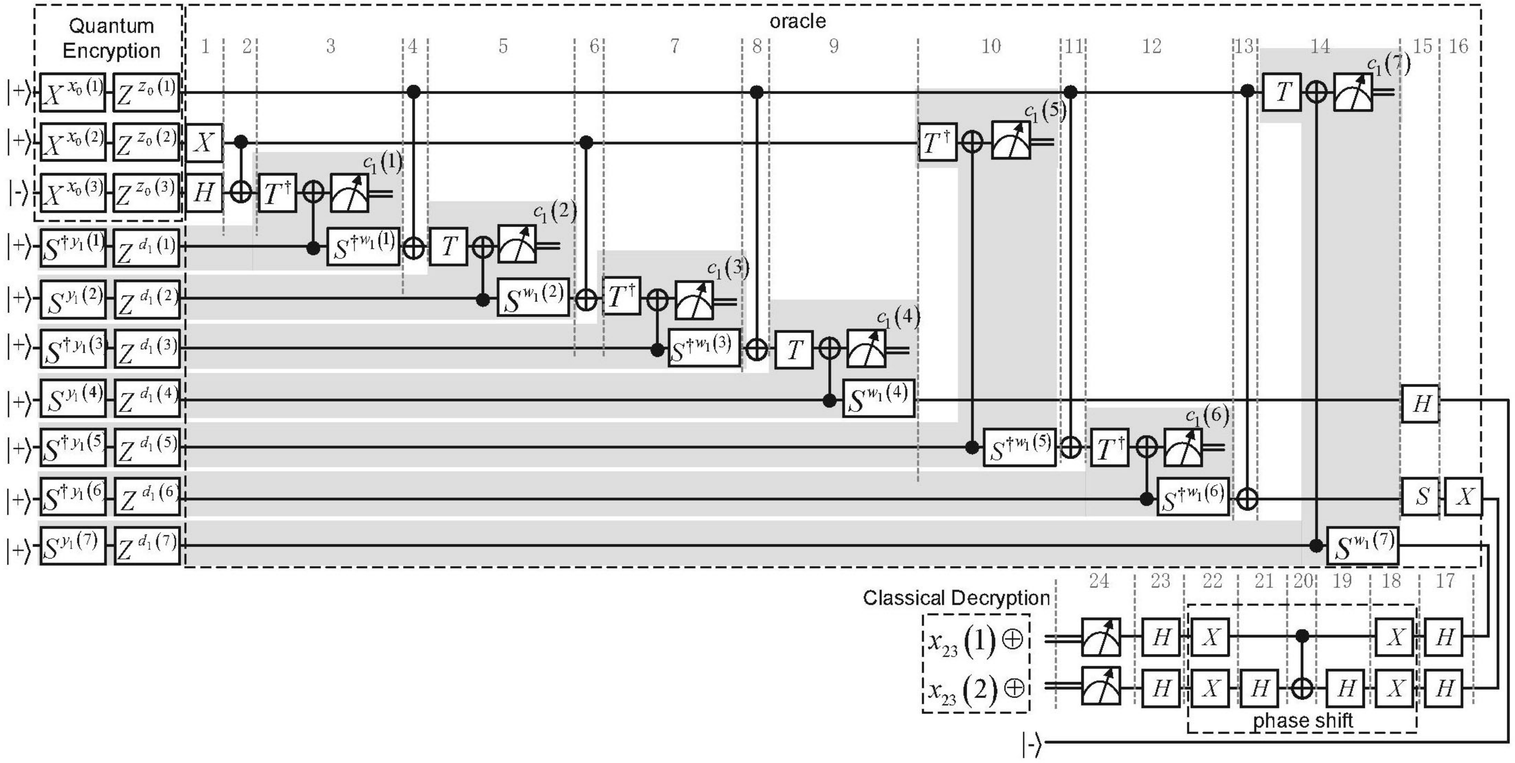

Figure S1. The quantum homomorphic search circuit for two-qubit encrypted states. The circuit can be divided into 24 steps (plus quantum encryption and classical decryption); each step corresponds to a round of key refresh. There are seven T (or $T^\dagger$) gadgets, each of which is marked by a shadowed area. The two-qubit input is encrypted with four key bits — $x_0(1), x_0(2), z_0(1)$ and $z_0(2)$, and the oracle qubit $|-\rangle$ can be thought of as being encrypted with key bits $x_0(3) = z_0(3) = 0$.
